# Effect of errors in pedigree on the accuracy of estimated breeding value for carcass traits in Korean Hanwoo cattle

**DOI:** 10.5713/ajas.19.0021

**Published:** 2019-10-21

**Authors:** Chiemela Peter Nwogwugwu, Yeongkuk Kim, Yun Ji Chung, Sung Bong Jang, Seung Hee Roh, Sidong Kim, Jun Heon Lee, Tae Jeong Choi, Seung-Hwan Lee

**Affiliations:** 1 Division of Animal and Dairy Science, Chungnam National University, Daejeon 34134, Korea; 2 Hanwoo Improvement Center, National Agricultural Cooperative Federation, Seosan 31948, Korea; 3 National Institute of Animal Science, Cheonan 31000, Korea

**Keywords:** Breeding Value, Carcass Traits, Genetic Gain, Hanwoo Cattle, Heritability

## Abstract

**Objective:**

This study evaluated the effect of pedigree errors (PEs) on the accuracy of estimated breeding value (EBV) and genetic gain for carcass traits in Korean Hanwoo cattle.

**Methods:**

The raw data set was based on the pedigree records of Korean Hanwoo cattle. The animals’ information was obtained using Hanwoo registration records from Korean animal improvement association database. The record comprised of 46,704 animals, where the number of the sires used was 1,298 and the dams were 38,366 animals. The traits considered were carcass weight (CWT), eye muscle area (EMA), back fat thickness (BFT), and marbling score (MS). Errors were introduced in the pedigree dataset through randomly assigning sires to all progenies. The error rates substituted were 5%, 10%, 20%, 30%, 40%, 50%, 60%, 70%, and 80%, respectively. A simulation was performed to produce a population of 1,650 animals from the pedigree data. A restricted maximum likelihood based animal model was applied to estimate the EBV, accuracy of the EBV, expected genetic gain, variance components, and heritability (*h*^2^) estimates for carcass traits. Correlation of the simulated data under PEs was also estimated using Pearson’s method.

**Results:**

The results showed that the carcass traits per slaughter year were not consistent. The average CWT, EMA, BFT, and MS were 342.60 kg, 78.76 cm^2^, 8.63 mm, and 3.31, respectively. When errors were introduced in the pedigree, the accuracy of EBV, genetic gain and *h*^2^ of carcass traits was reduced in this study. In addition, the correlation of the simulation was slightly affected under PEs.

**Conclusion:**

This study reveals the effect of PEs on the accuracy of EBV and genetic parameters for carcass traits, which provides valuable information for further study in Korean Hanwoo cattle.

## INTRODUCTION

Pedigrees are essential tools in the livestock breeding industry, because they provide ancestral information and knowledge for predicting progeny performance. A pedigree record contains the performance records of individuals and their progeny, and each domestic animal species has traits that are of economic value. For examples, meat and milk traits in cattle, sheep and goats [1]. Carmen [2] reported that pedigree was initially used in cattle breeding and other domestic animals. Henceforth, it becomes the principal breeding tool in the livestock sector. The importance of pedigree records in livestock breeding cannot be overemphasized, because the accuracy of selection depends on the superiority and size of the performance records that are available. Pedigree and performance records have been previously used for evaluation of genetic improvement and selection of animals with the highest genetic merit [3]. More so, the pedigree information can explain the genetic differences between and within individuals, creating an essential technique to evaluate parameters such as inbreeding, generation interval, estimated breeding value (EBV), heritability, and effective population size. These parameters could be utilized for proper selection and maintenance of healthy and genetically superior animals [4]. Pedigree and performance records have been earlier used to evaluate the genetic merit of animals in Hanwoo breeding scheme with proper selection of proven bulls [5]. Lee et al [6,7] also used Hanwoo pedigrees to identify the major loci associated with carcass weight (CWT) and intramuscular fat. On the other hand, Long et al [8] stated that the use of raw data for estimation of EBV of livestock could produce biased estimates when pedigree contains errors. In this case, these estimates would be an inaccurate genetic evaluation and slower genetic progress in that population.

Harder et al [9] described two types of pedigree errors (PEs), which could affect the EBV and genetic gain in a dairy cattle population. One of them is missing pedigree information (unknown parents), whereas the other is mistaken pedigree information (wrong parents). They added that, the proportion of wrong paternity decreased the estimates of genetic parameters. Previous studies by Israel and Weller [10], Christensen et al [11], and Gelderman et al [12] showed the consequences of PE or incorrect sire information in estimation of genetic parameters, for example, decreased value of parent transmitting ability for a cow and her relatives, reduced EBV, *h*^2^, and genetic gain for meat and milk traits in cattle populations. In addition, biased estimates of EBV and genetic gain for both bulls and cows have been reported [10,13–15]. Although, the effect of PEs on the accuracy of EBV and genetic estimates might not be available in Korean Hanwoo cattle. As a result, the knowledge of PEs on the accuracy of genetic parameters (EBVs, genetic gain, and *h*^2^) would be useful in the Hanwoo beef industry. Therefore, the aim of this study was to assess the effect of PEs on the accuracy of EBV and genetic gain for carcass traits.

## MATERIALS AND METHODS

### Raw data

The raw data set was based on the pedigree records of Korean Hanwoo cattle, which were the offspring of Korean proven bulls with different dam lines. The animals’ records were obtained using Hanwoo registration records from Korean animal improvement association database [16]. The record comprised of 46,704 animals, where the number of the sires used was 1,298 and the dams were 38,366 animals. The pedigree record consists of the performance and progeny test data sets of contemporary and ancestral relatives. The traits considered were CWT, eye muscle area (EMA), back fat thickness (BFT), and marbling score (MS). The measurements of carcass traits were in accordance with animal product grading service in South Korea [17]. Information about the production and breeding systems of Korean Hanwoo cattle are in accordance with Kim et al [18].

### Pedigree errors

Errors were introduced in the pedigree dataset through randomly assigning sires to all progenies born between 2000 and 2013. This method of changing sire records resulted in wrong sire information. SampleBy function in doBy package of the R software package was used for making PEs. For each generation, the pedigree dataset was substituted by error rates of 5%, 10%, 20%, 30%, 40%, 50%, 60%, 70%, and 80% as the true parent previously described in Oliehoek and Bijma [19].

### Simulated data

A simulation was performed to produce one replicate population of 1,650 animals using QMSim software package [20]. The number of sires was 150 whereas the dams were 1,500. For this replicate, the above-mentioned method of misidentification and error rates were introduced into the simulated data. More so, the effect of PE was assessed up to 12 generations with their averages. A single trait with a heritability of 0.4 and phenotypic variance of 2,071.47 were simulated in our study. The replacement ratios were 0.5 for sire and 0.4 for dam per generation.

### Statistical analyses

A restricted maximum likelihood (REML) based animal model was applied to estimate variance and covariance components of the studied traits using ASReml 4.0 software package [21]. The model included fixed effects of farm location (2), year of birth (3), and season of birth (7). A linear covariate of slaughter age was also fitted in the model. The mixed-model equation of the animal model used in the study was:

(1) $$\begin{array}{l} \text{y}=\text{Xb}+\text{Zu}+\text{e}\\ \text{and }var\;\left[\begin{array}{c} u\\ e \end{array}\right]=\left[\begin{array}{cc} A\sigma_{A}^{2} & 0\\ 0 & I\sigma_{E}^{2} \end{array}\right] \end{array}$$

where y, b, u, and e, are the vectors of phenotypes (lists of traits), fixed effects, random effects and residual errors, respectively, and X and Z are the design matrices.

### Breeding value estimation

The similar animal model was also used for estimation of breeding values using Henderson’s BLUP method [22] as implemented in ASReml 4.0. The accuracies of EBV estimation under different error levels of the pedigree were then calculated for all studied traits. Pearson’s correlation was used for each trait to assess the influence of PEs on the prediction of EBV.

### Expected genetic gain

The expected genetic gain from the selection was calculated using the following equation.

(2) $$\Delta G_{yr}=\frac{i\times r_{IH}\times G_{A}}{L}$$

where Δ*G_yr_* = expected genetic gain/yr, *i* = intensity of selection, *r_IH_* = accuracy, *G_A_* = genetic standard deviation, *L* = generation interval.

In the equation the term *i*, *L* were set as 0.80, 5.5, *r_IH_* = estimates in Table 3 per each trait, *G_A_* = square root of σ^2^_a_ per each trait. Then, the expected genetic gain per year for each trait was obtained from the studied dataset.

### Estimates of variances and heritability

The variance components as well as *h*^2^ estimates for carcass traits were estimated using a single trait animal model in equations (1), whereas the equation for *h*^2^ estimates was as follows,

(3) $$h^{2}={\sigma^{2}}_{\text{a}}/{\sigma^{2}}_{\text{p}}$$

where σ^2^_a_, additive genetic variance; σ^2^_p_, phenotypic variance; *h*^2^, heritability.

## RESULTS

The average carcass traits per year are presented in Table 1. All the traits showed inconsistency between the years. The CWT, EMA, BFT, and MS showed an increase by 65.61 kg, 4.96 cm^2^, 1.76 mm, and 0.79, respectively. Table 2 illustrates the overall mean, standard deviation, minimum, maximum and coefficient of variation for carcass traits. The average values for CWT, EMA, BFT, and MS were 342 kg, 78.76 cm^2^, 8.63 mm, and 3.31, respectively.

**Figure 1:**
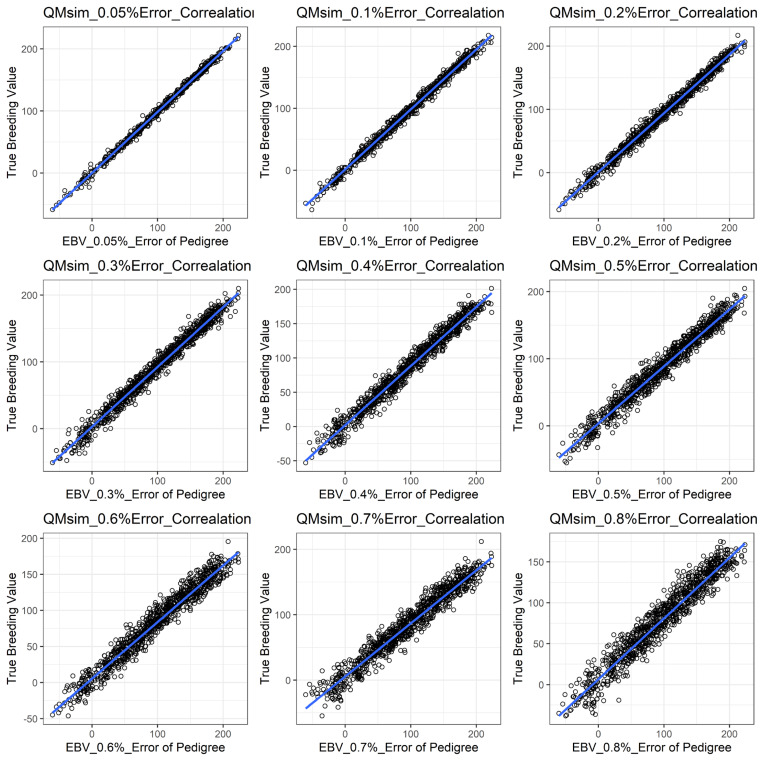
The accuracy of EBV of carcass trait for simulated data under pedigree error scenario. This indicates that the accuracy of EBV of a carcass trait for simulated data slightly declined with increased error rates. EBV, estimated breeding value.

**Figure 2:**
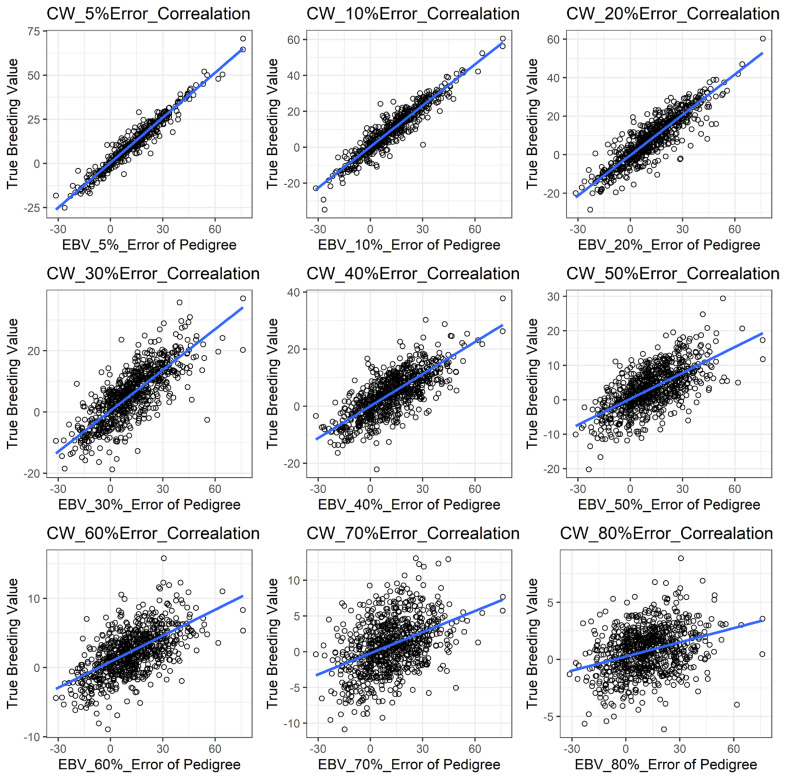
The accuracy of EBV for CWT under different pedigree error rates. This illustrates the negative effect of error in pedigree on the accuracy of EBV for CWT. The accuracy of EBV for CWT continues to decrease with increased errors. EBV, estimated breeding value; CWT, carcass weight.

**Figure 3:**
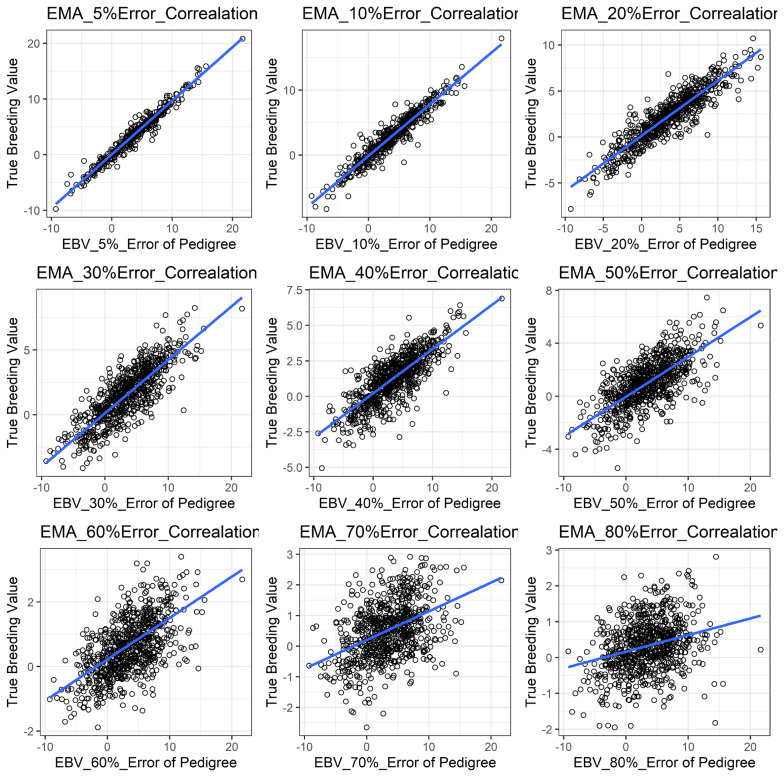
The accuracy of EBV for EMA under different pedigree error rates. This illustrates the negative effect of error in pedigree on the accuracy of EBV for EMA. The accuracy of EBV for EMA continues to decrease with increased errors. EBV, estimated breeding value; EMA, eye muscle area.

**Figure 4:**
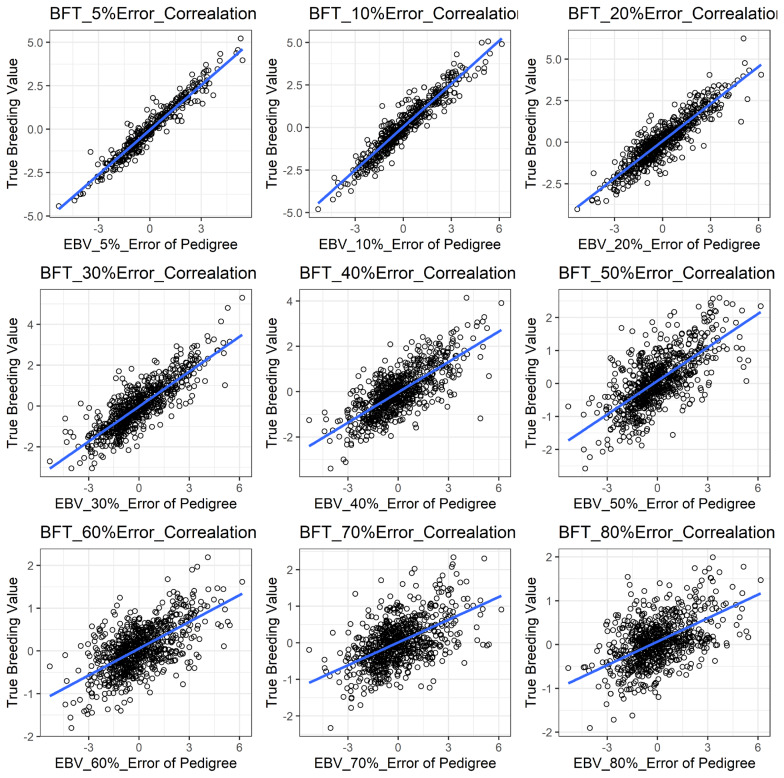
The accuracy of EBV for BFT under different pedigree error rates. This illustrates the negative effect of error in pedigree on the accuracy of EBV for BFT. The accuracy of EBV for BFT continues to decrease with increased errors. EBV, estimated breeding value; BFT, back fat thickness.

**Figure 5:**
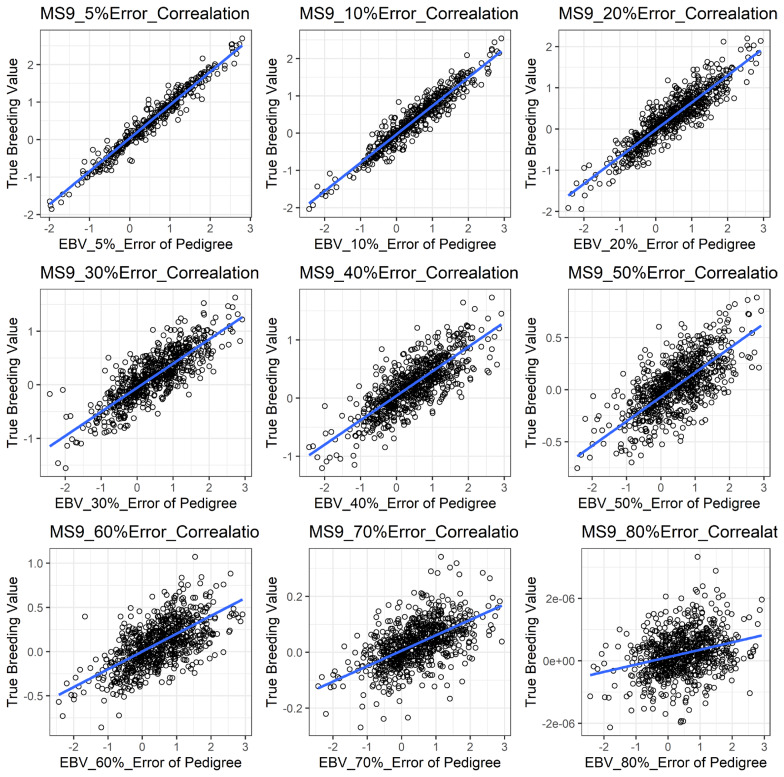
The accuracy of EBV for MS under different pedigree error rate. This illustrates the negative effect of error in pedigree on the accuracy of EBV for MS. The accuracy of EBV for MS continues to decrease with increased errors. EBV, estimated breeding value; MS, marbling score.

Figure 1 shows the accuracy of EBV for simulated data under PE scenario. The graphical presentations of the accuracy of EBVs for each trait under PE situation with raw data are shown in Figure 2 to 5. Table 3 indicates the EBV accuracy for carcass traits under PE scenario, and the result shows that all the studied traits were affected by errors. The correlations of the simulated data under PE are presented in Table 4. The result indicates that, the correlation of the carcass trait was slightly decreased as errors were introduced in the pedigree.

Table 5 shows the expected genetic gain for carcass traits. In this study, the expected genetic gain for carcass traits were 3.13 kg, 0.80 cm^2^, 0.34 mm, and 0.17 for CWT, EMA, BFT, and MS with no PE (0%), and that deemed to decline constantly as PEs were increased gradually in the dataset. With a 5% PE, the decline in the traits were 0.34 kg, 0.03 cm^2^, 0.01 mm, and 0.01 for CWT, EMA, BFT, and MS with respect to that estimate at 0% PE. This result was followed by the highest values of 2.85 kg, 0.73 cm^2^, 0.26 mm, and 0.17 decline at 80% PE.

Table 6 presents the variance components and *h*^2^ estimates for carcass traits. For CWT, the estimated *h*^2^ with no PE was 0.36 in this study. However, this *h*^2^ decreased consistently as more PE introduced in the dataset and reached to as low as 0.03 at 80% of PE. A very similar negative effect of PE on *h*^2^ of EMA was observed as well, where *h*^2^ with no error was estimated as high as 0.42 and as low as 0.05 at 80% PE. The presence of PE equally affected the *h*^2^ of BFT and MS. In this regard, BFT *h*^2^ was reduced from 0.48 with no PE to 0.11 with 80% of PE, whereas for MS, such decrease in *h*^2^ was from 0.58 to a negligible heritability (0.00).

## DISCUSSION

In this study, we assessed the effect of PE on the accuracy of EBVs, genetic gain, and *h*^2^ estimates of carcass traits. In general, the carcass traits per year were evaluated from 2000 to 2013 as shown in Table 1, which shows overall improvements in most of the traits. Although, increased value of carcass traits was not consistent between the years. Park et al [23] reported similar observations on carcass traits between 1998 and 2012. In addition, a comparison of carcass traits between 2000 and 2013 indicated an increase in our study.

The overall mean of carcass traits is presented in Table 2. Park et al [23] and Yoon et al [24] reported similar results for carcass traits previously in Hanwoo Korean cattle. More recently, Do et al [25] reported slightly higher values for carcass traits in Hanwoo cattle compared with our study. On the other hand, lesser values in some of the carcass traits have been observed [26] in Japanese black (Wagyu). Additionally, we need to consider the influences from the breed under study or genotype-environment interactions on animals that could introduce variations in growth and carcass traits, as suggested earlier by Fabrizio et al [27].

With a dataset including PE, some parameters were found to be influenced in our study (Table 3). We observed that PE had negative associations with the accuracy of EBV of the studied traits. In this case, more errors in the pedigree also reduced the evaluation accuracy in those traits noticeably and that could be a great disadvantage to selection responses in the breeding program. Our results are also in accordance with Israel [10], Ron et al [13], and Bovenhuis and Van Arendonk [28] that similarly reported lower accuracy in EBV with the presence of errors in the pedigree. All traits in this study showed similar trends with respect to PE, even though there were differences in magnitudes of influences on each trait evaluation. Long et al [8] in this regard, agreed with our results by showing a reduction in accuracy of EBV for litter size, BFT and average daily gain in swine. Banos et al [29] also reported a 9% milk yield decrease in bull due to PE in their study.

The correlations of the simulated data under PE were slightly reduced in our study as shown in Table 4. Our results indicated that the influence of PE on the correlation was somewhat lower. This is probably because our simulated population was not as complicated as the raw dataset. Similar reductions in response between simulated BV and EBV were observed by the study of Van Arendonk et al [30].

We found that the expected genetic gain in animals was also largely reduced due to PEs as presented in Table 5. Our estimated genetic gain and its reduction due to PE is also comparable to other studies by Long et al [8], Israel [10], Christensen et al [11], and Bovenhuis and Van Arendonk [28]. A report on reduced genetic gain, as Angeln dairy cattle population by Sanders et al [31], due to either erroneous or missing sire information was also in agreement with this study. Van Arendonk et al [30] also reported lower genetic gain in a closed pig-breeding nucleus with introduction of errors in the pedigree.

The variance components and *h*^2^ estimates for carcass traits are illustrated in Table 6. For all the studied traits, the *h*^2^ estimates were negatively affected as more errors were introduced in the pedigree. The *h*^2^ being affected by PE also indicated that such errors could reduce the selection accuracy at the same time. For this reason, *h*^2^ is very important to selection for polygenic traits, because selection accounts for those animals with the best breeding values to become the parents of the next generation. In order to increase selection accuracy, we need good information about the candidates for selection because the only information available is the phenotypic records, which is the strength of the relationship between phenotypic values and breeding values (i.e., *h*^2^). Therefore, when *h*^2^ is low, the phenotypic values mostly reveal little about the underlying breeding values, and it is difficult to determine which animals have the best breeding values to become the potential parents [32]. Our study is also comparable to those reported earlier by Senneke et al [33] who observed a reduction in *h*^2^ estimates for birth and weaning weights in Herford cattle. Previous studies by Gelderman et al [12], and Parlato and Van Vleck [34] also reported decreased *h*^2^ estimates in milk fat and milk yield in both cattle and buffalo populations under erroneous pedigree.

## CONCLUSION

The accuracy of EBV, genetic gain, and heritability estimates for the studied traits were affected by introduction of errors in the pedigree. On the other hand, the result of the correlation estimates of simulated data for carcass trait was slightly decreased as errors were introduced in the pedigree. As a result, PEs had a negative effect on the overall estimates, which could slower the rate of genetic or selection progress in a population.

## Figures and Tables

**Table 1:** Average phenotypic values for carcass traits per year

Year	CWT (kg)	EMA (cm2)	BFT (mm)	MS9
2000	313.37	75.61	7.84	-
2001	307.69	75.54	6.90	-
2002	324.43	74.33	8.06	-
2003	352.04	75.72	10.59	3.57
2004	356.07	78.63	10.49	3.21
2005	360.36	76.87	10.46	3.21
2006	358.18	79.65	10.60	3.33
2007	361.63	82.10	9.32	3.54
2008	360.31	84.16	8.16	3.13
2009	354.05	82.03	7.94	3.04
2010	361.45	83.91	8.27	3.03
2011	362.73	81.81	8.47	3.14
2012	359.32	79.58	8.21	3.32
2013	378.98	80.57	9.60	4.36

CWT, carcass weight; EMA, eye muscle area; BFT, back fat thickness; MS9, marbling score with 9 levels.

**Table 2:** Basic statistics for carcass traits

Trait	Mean	SD	Min	Max	CV
CWT	342.60	45.51	158	518	0.13
EMA	78.76	9.23	22	123	0.12
BFT	8.63	3.71	1	35	0.43
MS	3.31	1.61	1	9	0.49

SD, standard deviation; Min, minimum; Max, maximum; CV, coefficient of variation; CWT, carcass weight; EMA, eye muscle area; BFT, back fat thickness; MS9, marbling score with 9 levels.

**Table 3:** Accuracy of estimated breeding value for carcass traits under different error levels

Pedigree error (%)	CWT	BFT	EMA	MS
5	0.96	0.96	0.97	0.97
10	0.94	0.94	0.95	0.95
20	0.90	0.90	0.91	0.89
30	0.77	0.83	0.81	0.81
40	0.75	0.73	0.78	0.78
50	0.63	0.68	0.69	0.70
60	0.57	0.62	0.56	0.61
70	0.40	0.53	0.38	0.55
80	0.30	0.52	0.24	0.26

CWT, carcass weight; BFT, back fat thickness; EMA, eye muscle area; MS9, marbling score with 9 levels.

**Table 4:** Correlation estimates of simulated data under pedigree error

Pedigree error (%)	Correlation coefficient
5	0.99
10	0.99
20	0.99
30	0.98
40	0.98
50	0.98
60	0.97
70	0.97
80	0.96

**Table 5:** Estimates of expected genetic gain for carcass traits under different pedigree error levels

Pedigree error (%)	CWT	BFT	EMA	MS
0	3.13	0.80	0.34	0.17
5	2.79	0.77	0.33	0.16
10	2.60	0.69	0.31	0.14
20	2.39	0.58	0.28	0.13
30	1.70	0.42	0.24	0.10
40	1.56	0.36	0.18	0.08
50	1.13	0.35	0.15	0.05
60	0.71	0.17	0.10	0.05
70	0.54	0.12	0.10	0.02
80	0.28	0.07	0.08	0.00

CWT, carcass weight; BFT, back fat thickness; EMA, eye muscle area; MS9, marbling score with 9 levels.

**Table 6:** Estimates of variance components and heritability for carcass traits under different pedigree error levels

Pedigree error (%)	CWT				EMA				BFT				MS			
			
	*σ*^2^_a_	*σ*^2^_e_	*σ*^2^_p_	*h*^2^	*σ*^2^_a_	*σ*^2^_e_	*σ*^2^_p_	*h*^2^	*σ*^2^_a_	*σ*^2^_e_	*σ*^2^_p_	*h*^2^	*σ*^2^_a_	*σ*^2^_e_	*σ*^2^_p_	*h*^2^
0	463.06	814.05	1,277.12	0.36	30.33	41.44	71.77	0.42	5.77	6.22	11.98	0.48	1.44	1.04	2.48	0.58
5	393.43	863.68	1,257.11	0.31	29.96	39.98	69.95	0.42	5.57	6.45	12.02	0.46	1.29	1.17	2.47	0.52
10	358.03	897.50	1,255.53	0.28	24.69	45.02	69.71	0.35	5.11	6.88	11.98	0.43	1.09	1.37	2.46	0.44
20	330.96	923.02	1,253.98	0.26	19.43	50.09	69.53	0.27	4.69	7.29	11.98	0.39	1.02	1.44	2.47	0.41
30	227.72	1,020.73	1,248.45	0.18	13.07	55.98	69.05	0.18	3.89	8.08	11.98	0.32	0.71	1.74	2.45	0.29
40	201.76	1,047.09	1,248.85	0.16	10.35	58.58	68.93	0.15	3.09	8.80	11.89	0.25	0.61	1.83	2.44	0.25
50	150.56	1,094.74	1,245.29	0.12	12.43	56.74	69.18	0.17	2.32	9.52	11.84	0.19	0.32	2.10	2.42	0.13
60	74.47	1,167.48	1,241.95	0.05	4.59	63.95	68.54	0.06	1.39	10.41	11.81	0.12	0.33	2.09	2.42	0.13
70	86.44	1,155.89	1,242.33	0.06	4.69	63.83	68.52	0.06	1.75	10.08	11.83	0.15	0.07	2.33	2.41	0.03
80	42.60	1,198.27	1,240.87	0.03	3.75	64.76	68.51	0.05	1.29	10.50	11.79	0.11	0.00	2.41	2.41	0.00

*σ*^2^_a_, additive variance; *σ*^2^_e_, residual variance; *σ*^2^_p_, phenotypic variance; *h*^2^, heritability.
